# B7-Positive and B7-Negative Acute Myeloid Leukemias Display Distinct T Cell Maturation Profiles, Immune Checkpoint Receptor Expression, and European Leukemia Net Risk Profiles

**DOI:** 10.3389/fonc.2020.00264

**Published:** 2020-03-13

**Authors:** Ion Antohe, Angela Dǎscǎlescu, Cǎtǎlin Dǎnǎilǎ, Amalia Titieanu, Mihaela Zlei, Iuliu Ivanov, Adriana Sireteanu, Mariana Pavel, Petru Cianga

**Affiliations:** ^1^Hematology Department, Grigore T. Popa University of Medicine and Pharmacy, Iaşi, Romania; ^2^Hematology Department, Regional Oncology Institute, Iaşi, Romania; ^3^Immunophenotyping Department, Regional Oncology Institute, Iaşi, Romania; ^4^Molecular Diagnostic Department, Regional Oncology Institute, Iaşi, Romania; ^5^Immunology Department, Grigore T. Popa University of Medicine and Pharmacy, Iaşi, Romania

**Keywords:** acute myeloid leukemia (AML), immunotherapy, checkpoint ligand, B7 molecules, programmed death 1 (PD-1), inducible T cell costimulator (ICOS)

## Abstract

Acute myeloid leukemia (AML) is generally considered a poorly immunogenic malignancy, displaying a “non-inflamed” leukemia microenvironment (LME), leading to T cell tolerance. However, the immune landscape of AML is much more heterogeneous. Since B7 expression is regarded as a consequence of an interferon-mediated “inflammatory” phenotype, we have investigated by flow cytometry the B7 checkpoint ligands B7.1, B7.2, programmed death ligand 1 (PD-L1), PD-L2, ICOS-L, B7-H3, and B7-H4 on the AML blasts of 30 newly diagnosed patients and their corresponding receptors [cytotoxic T lymphocyte-associated protein 4 (CTLA-4), programmed death 1 (PD-1), and inducible T cell costimulator (ICOS)] on bone marrow (BM) T cell maturation populations. We could thus evidence B7-negative and B7-positive leukemias either with an isolated expression or part of eight different checkpoint ligand “signatures” that always included an inhibitory B7 molecule. B7-positive AMLs encompassed intermediate and adverse European Leukemia Net (ELN) risk cases and displayed mainly central memory CD4+ T cells with high ICOS levels and effector CD8+ T cells with high PD-1 expression. B7-negative cases were rather classified as AML with recurrent genetic anomalies and displayed predominantly naive T cells, with the exception of *NPM1* mutated AMLs, which expressed B7-H3. These different B7 immune profiles suggest that specific immunotherapies are required to target the distinct immune evasion strategies of this genetically heterogeneous disease.

## Introduction

The last decade has witnessed dramatic advances in the field of cancer immunotherapy. Immune checkpoint blockade (ICB) is reshaping the treatment paradigm of solid tumors (1) and hematologic cancers, such as Hodgkin lymphoma (2). Furthermore, CD19-directed chimeric antigen receptor (CAR)-T cells and the bispecific T cell engager (BiTE®) blinatumomab have produced spectacular remissions in acute lymphoblastic leukemia and diffuse large B cell lymphoma (3–6).

Simultaneously, gene expression profiling (GEP) of tumor immune microenvironments is revolutionizing our understanding of cancer-immune interactions. Several recurrent pan-cancer immune profiles have been identified and could serve as biomarkers for predicting clinical responses to immunotherapy or for tailoring personalized treatment strategies (7, 8). Briefly, tumors with inflamed type I and II interferon (IFN)-driven immune microenvironments (informally designated as “hot”) are ICB responsive, while “cold” “immune-desert” tumors would rather benefit from adoptive cell transfer or tumor–peptide vaccination (9).

The development of immunotherapy in acute myeloid leukemia (AML) has been hindered so far not only by its remarkable genetic, antigenic, and clonal heterogeneity (10–13) and the risk of significant off-target hematologic toxicity but also by the lack of biomarkers defining patient populations more likely to benefit from it (9). Recent research revealed that immune profiles are identifiable in human AML and hold prognostic and therapeutic relevance (9, 14). The AML immune response shares numerous traits with solid cancers (15) and offers various opportunities for immunotherapy (9). However, given its origin within an immune-privileged, regulatory T cell (Treg)-abundant bone marrow (BM) niche (16), its low mutational load (11), deficient antigen presentation (17–20), aggressive growth, and bloodstream dissemination (21, 22), AML was regarded as a poorly immunogenic tumor with a rather “immune-desert” leukemia microenvironment (LME) phenotype (9), in which immunoediting is either absent or a rather late event (23).

Noteworthy, the LME of some AML cases displays evidence of a prior antileukemic immune response, restrained by immune escape mechanisms such as cytotoxic T lymphocyte associated protein-4 (CTLA-4)/B7.1/B7.2 and programmed death 1 (PD-1)/PD-1L signaling or Treg expansion (9). B7 molecules are key structures of the immune checkpoints that regulate T cell activation (24). Previous research has shown that upregulation of B7 ligands such as programmed death ligand 1 (PD-L1), PD-L2, and B7.2 on AML blasts is inducible by exposure to interferon γ (IFN-γ) (25–27), which indicates an “inflamed” immune profile. Hijacking of these immune checkpoint molecules is used by leukemia cells to evade immune surveillance (28).

The expression of IFN-γ-responsive genes has been correlated with primary refractory AML and is able to predict responses to ICB or flotetuzumab (CD3/CD123 DART®), showing that immune signatures within the LME can serve as reliable biomarkers to predict responses to immunotherapy (14, 29).

Previous research was rather focused on finding prognostic relevance of isolated B7 molecule expression in AML (28, 30–32), and only very recently a comprehensive analysis of B7 checkpoint ligand co-expression correlated with checkpoint receptors and T cell populations was conducted (33).

Well-beyond investigating the expression of isolated molecules, our study aims to simultaneously evaluate the B7 checkpoint ligand phenotype of AML blasts (B7.1, B7.2, PD-L1, PD-L2, ICOS-L, B7-H3, B7-H4) and the expression of immune checkpoint receptors (ICRs) [inducible T cell costimulator (ICOS), PD-1, CTLA-4] on helper and cytotoxic T cell maturation populations and to correlate these data to standard prognostic factors. We advanced the notion of “B7 checkpoint ligand signatures” to systematize the co-signaling output of AML blasts toward T cells. Since the expression of the immune escape PD-1/PD-L1 axis is correlated with an IFN-rich “inflamed” LME (9), it will be a challenge for future studies to demonstrate that the expression of B7 checkpoint ligands could serve as feasible alternative or complementary markers of AML with “inflamed” microenvironment, impacting upon distinctive immunotherapy approaches.

## Materials and Methods

### Patient Selection

Based on informed consent, 30 patients diagnosed with *de novo*, non-promyelocytic AML between 2016 and 2019 at the Iaşi Regional Oncology Institute, Romania, were included in this study. This study has been approved by the institutional ethics committee. BM and peripheral blood (PB) samples were collected at diagnosis. AML diagnosis was established according to the WHO diagnostic criteria (34), and patients were risk-stratified in accordance with the 2017 European Leukemia Net (ELN) recommendations (35). We have also analyzed the BM and PB samples of four healthy volunteers after their informed consent.

### Flow Cytometry

AML blasts and T cells were analyzed by multiparameter flow cytometry (MFC) on erythrocyte-lysed fresh BM and PB samples. This study comprised four phases: (1) confirmation of AML diagnosis with EuroFlow standardized monoclonal antibody (MoAbs) panels (2, 36) analysis of the expression of B7-1, B7-2, PD-L1, B7-H2, PD-L2, B7-H3, B7-H4 on AML blasts; (3) analysis of Treg percentages and T cell maturation subsets in the BM; (4) evaluation of PD-1, ICOS, and CTLA-4 expression on T cell maturation subsets. In line with previous research regarding B7 checkpoint ligand expression in AML but also solid cancers, B7 molecules were considered positive if present on more than 10% of the total AML cells (30, 32, 37).

Data acquisition was performed on a BD FACS ARIA III cytometer, and data were interpreted using the FACS DIVA v6.1.3 software. An identical investigation protocol was applied for all healthy subjects. The MoAbs used in this study are detailed in Supplementary Table 1.

AML blasts gating was performed on CD45+/CD34+/CD117+/HLA-DR+ events. Subsequently, the expression level of each B7 molecule was assessed.

T cell gating was performed on CD3+/CD4+ and CD3+/CD8+ events. The following T cell maturation subsets were defined based on their differential expression of CD28, CD27, and CD45RA: naive (N), central memory (CM), intermediate effector memory (iEM), late effector memory (late EM) (38, 39). Tregs were defined as CD3+/CD4+/CD25+/CD127- events (40). A T cell population was considered predominant if it outnumbered each of the other T cell subsets. Finally, the expression of ICOS, PD-1, and CTLA-4 was evaluated on BM total and maturation subsets of CD4+ and CD8+ cells. All the experiments were performed in compliance with the rules of standard biosecurity and institutional safety procedures.

### Statistical Analysis

Statistical analyses were performed using the IBM® SPSS Statistics 21.0 Software. Each figure contains the relevant statistical information: the *n*, total number of patients, the significance *p*-value, the statistical test used. The chi square, Fisher's exact test, Mann–Whitney test, and Student's *t*-test were used to analyze the associations between different variables. The Pearson correlation coefficient was calculated to investigate the relationships between numerical variables. The two-way ANOVA test was used to analyze the differences among multiple variables. A *p* < 0.05 was considered as statistically significant.

## Results

### Baseline Patient Characteristics

Patient data are summarized in Table 1. The median age at diagnosis was 59 years (range 27–83 years). A total of 23.3% of patients had favorable ELN risk cytogenetics, 46.7 and 30% had intermediate and, respectively, adverse karyotypes. Two patients harbored *FLT-3-ITD* mutations, and three patients had *NPM1* mutated status. *FLT-3-ITD* and *NPM-1* mutations did not coexist in our study group.

**Table 1:** Patterns of expression of B7 ligands, ICRs, T cell populations relative to WHO AML type and ELN risk.

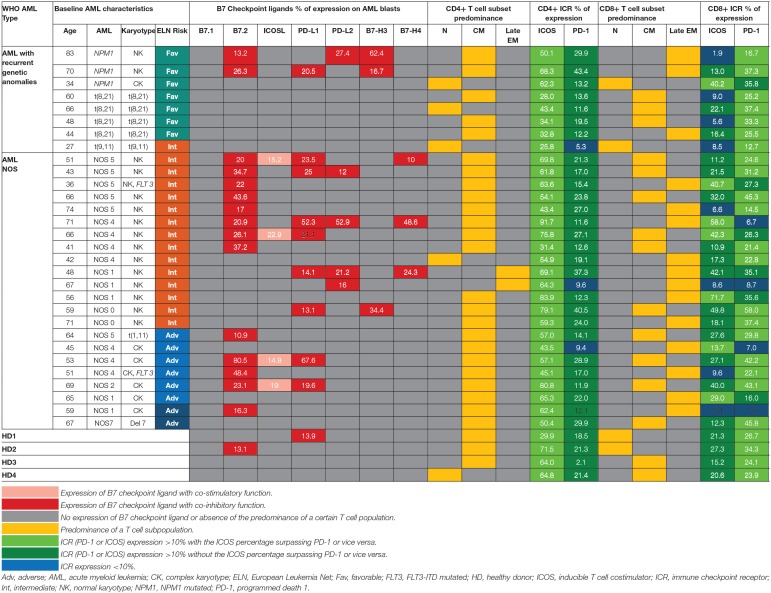

### B7 Checkpoint Ligands Are More Frequently Expressed in Intermediate and Adverse Risk Acute Myeloid Leukemia

**Figure 1:**
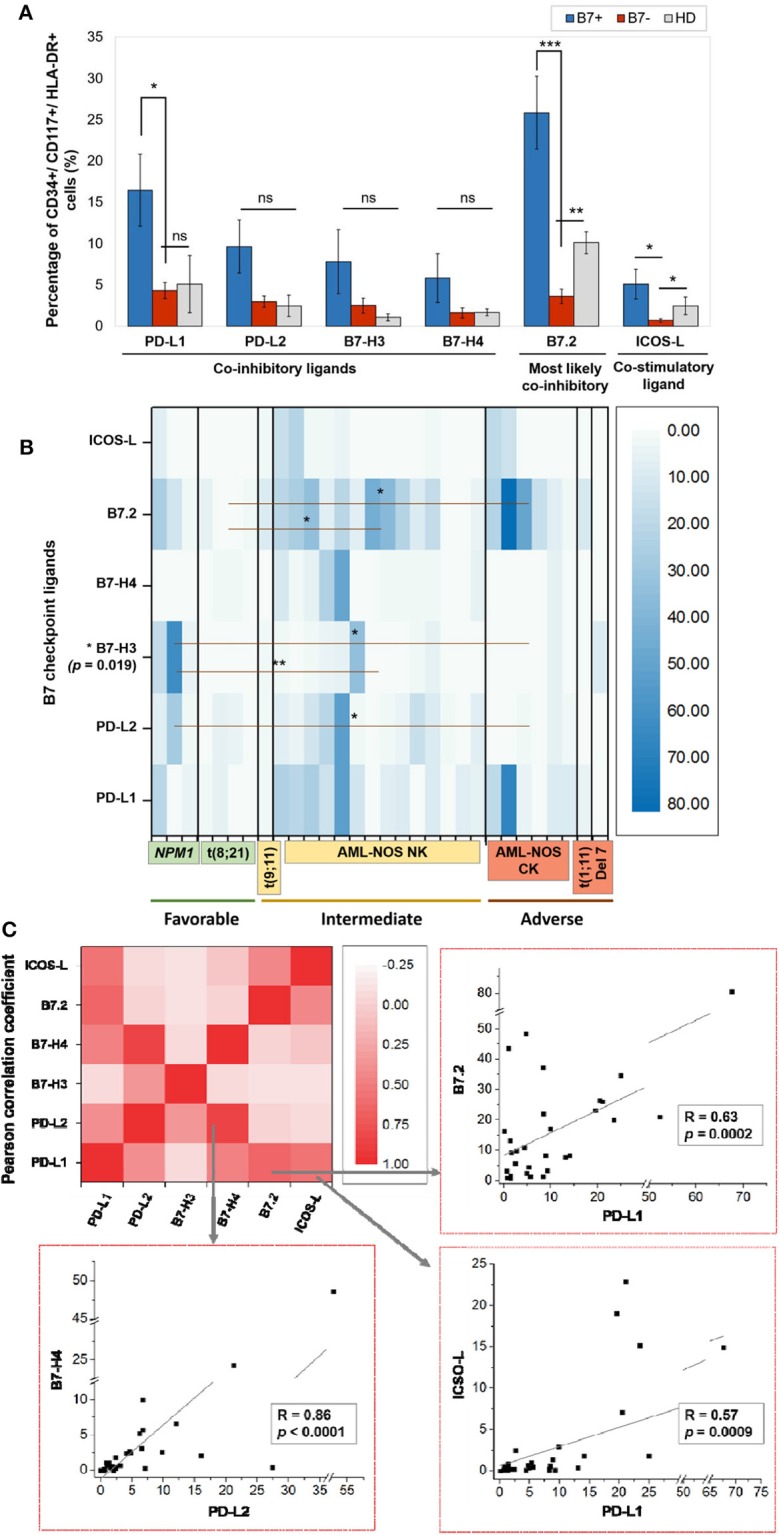
**(A)** Percentage of CD34+/CD117+/HLA-DR+ cells expressing the indicated B7 checkpoint ligands in patients, categorized as B7+ (*n* = 18) or B7– (*n* = 12) based on the B7 ligand expression and healthy donors (HD, *n* = 4). Bars represent the mean ± SEM (***P < 0.001, **P < 0.01, *P < 0.05; ns, not significant; two-tailed *t*-test, Mann–Whitney test). **(B)** Heat map of percentages of acute myeloid leukemia (AML) blasts expressing the indicated B7 checkpoint ligands in patients categorized based on their corresponding European Leukemia Net (ELN) risks: favorable, intermediate, and adverse (**P < 0.01, *P < 0.05; two-tailed *t*-test). **(C)** Heat map of B7 ligand co-expression. Pearson correlation coefficients (*R*) were computed, and ANOVA test was used to validate the significance of the identified correlations.

Within the healthy donor (HD) group, we could evidence the isolated expression of two molecules, PD-L1 and B7.2, in two cases (Table 1), while 18 patients (60%) were identified with B7 ligand expression. The B7 molecule levels differed markedly from those of HD (Figure 1A): PD-L1 (B7+ vs. B7–: *p* = 0.028, B7– vs. HD: *p* = 0.739), B7.2 (B7+ vs. B7–: *p* = 0.0003, B7– vs. HD: *p* = 0.7111) and ICOS-L (B7+ vs. B7–: *p* = 0.049, B7– vs. HD: *p* = 0.011). Out of the B7-positive cases, 10 expressed B7 molecule signatures and eight had isolated B7 expression.

B7.2 was the most frequently expressed molecule (50% of cases, *n* = 15), followed by PD-L1 (30%, *n* = 9), PD-L2 (15%, *n* = 5), ICOS-L (12%, *n* = 4), B7-H3 (10%, *n* = 3), and B7-H4 (10%, *n* = 3). B7.1 was expressed at extremely low levels and was thus considered negative. B7.2 was equally expressed isolated or co-expressed, while all the other B7 ligands were mainly co-expressed on AML blasts as B7 signatures.

The majority of B7-positive patients (16 out of 18, 88.8%) had intermediate or adverse ELN risk AML-Not Otherwise Specified (NOS). Out of these, complex karyotype AML-NOS expressed either B7.2 isolated or co-expressed B7.2, ICOS-L, and PD-L1, while normal karyotype AML-NOS also expressed PD-L2, B7-H3, and B7-H4 alongside B7.2, ICOS-L, and PD-L1 (Figure 1B). The two *FLT-3 ITD* mutated, normal karyotype AML-NOS cases displayed only an isolated B7.2 expression (Table 1).

By contrast, favorable risk AML rarely expressed B7 molecules (2 out of 7 cases, 28.6%). Out of this group, *NPM1* mutated AML was the only B7-positive subtype and was correlated with B7-H3 expression (*p* = 0.02) and significantly higher levels of B7-H3 when compared to the AML-NOS cases (*p* = 0.019). AML with t (8,21)(q22;q22) was B7 negative and expressed significantly lower percentages of B7.2 (*p* = 0.036) when compared to the AML-NOS cases (Figure 1B).

Further details regarding the expression of each B7 checkpoint ligand relative to age, WHO AML type, and ELN risk are provided in Table 1. We found no significant correlation between patient age, gender, hyperleukocytosis, and the expression of B7 checkpoint ligands. However, B7 positivity was correlated with the presence of refractory AML (*p* = 0.017, chi square test) and worse overall survival (*p* = 0.004, log rank test) (data not shown).

### B7-Positive Leukemias Rather Express Inhibitory B7 Ligands

We have identified eight different B7 checkpoint ligand signatures in 10 patients (Table 1): co-expression of B7.2, ICOS-L, PD-L1 (three cases); B7.2, ICOS-L, PD-L1, B7-H4 (one case); B7.2, PD-L1, PD-L2 (one case); B7.2, PD-L1, B7-H3 (one case); B7.2, PD-L2, B7-H3 (one case); PD-L1; B7-H3 (one case); B7.2, PD-L1, PD-L2, B7-H4+ (one case); PD-L1, PD-L2, B7-H4+ (one case). A mean number of three B7 ligands were co-expressed in these signatures. PD-L1 and B7.2 were regularly expressed in B7 ligand signatures (90 and 80%, respectively), and all signatures included at least one B7 molecule with clearly defined or most likely co-inhibitory role, such as B7.2 (41).

Furthermore, we found statistically significant correlations between the expression levels of the following B7 ligand combinations: B7.2–PD-L1 (*p* = 0.0002), B7.2–ICOS-L (*p* = 0.019), PD-L1–ICOS-L (*p* = 0.0009), PD-L1–B7-H4 (*p* = 0.0051), PD-L2–B7-H4 (*p* < 0.0001). B7.2 expression was rather associated with ICOS-L and PD-L1 in the B7.2/PD-L1/ICOS-L (three cases) and B7.2/PD-L1/ICOS-L/B7-H4 signatures (one case). Moreover, PD-L2 was correlated to B7-H4 expression (*p* < 0.0001). However, B7.2 expression was not correlated to PD-L2, B7-H3, and B7-H4. Finally, B7-H3 and B7-H4 expression was mutually exclusive (*p* = 0.027; Figure 1C).

### Helper and Cytotoxic T Cells From Acute Myeloid Leukemia Patients Display Different Maturation and Immune Checkpoint Receptor Expression Patterns

**Figure 2:**
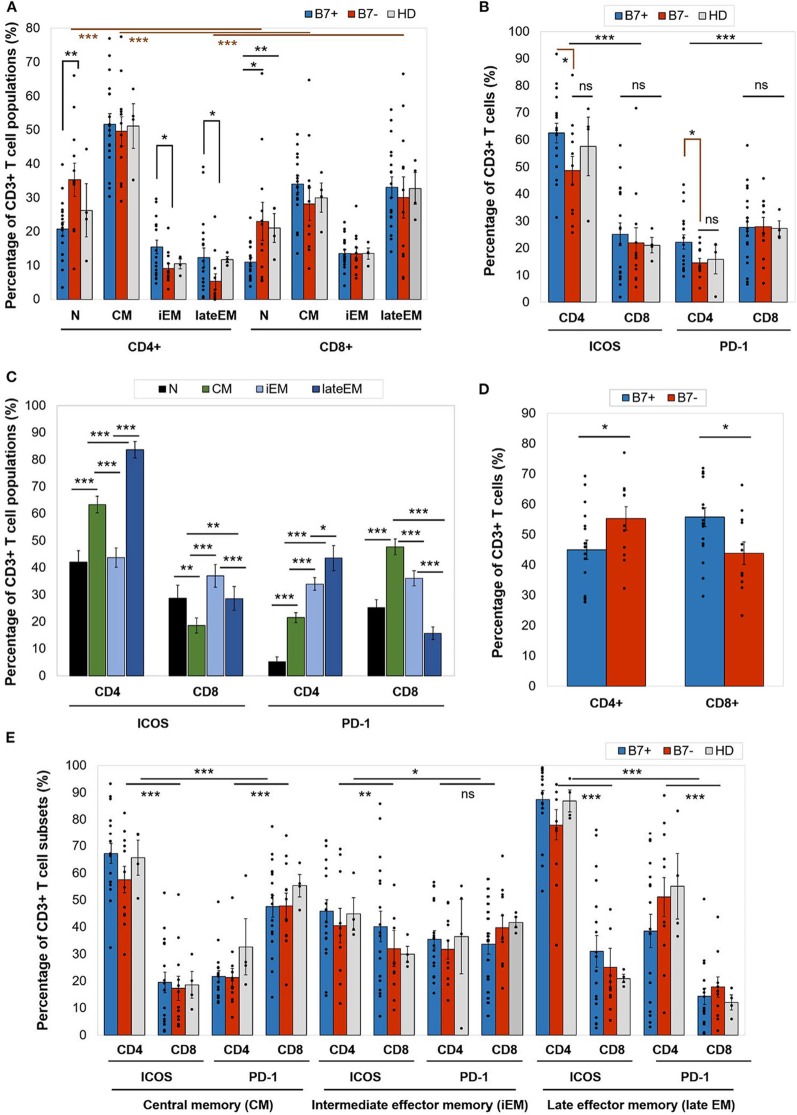
**(A)** Percentage of CD4+ or CD8+ T cell populations—naive (N), central memory (CM), intermediate effector memory (iEM), and late effector memory (late EM)—in AML patients (either B7+, *n* = 18, or B7–, *n* = 12) and healthy donors (HD, *n* = 4). Individual values are represented as points. Bars represent the mean ± SEM (**P < 0.01, *P < 0.05; two-tailed *t*-test, Mann–Whitney test). **(B)** Expression of immune checkpoint receptors [inducible T cell costimulator (ICOS) and programmed death 1 (PD-1)] on CD4+ or CD8+ T cells in AML patients (either B7+, *n* = 18, or B7-, *n* = 12) and healthy donors (HD, *n* = 4). Individual values are represented as points. Bars represent the mean ± SEM (***P < 0.001, *P < 0.05; ns, not significant; two-tailed *t*-test and two-way ANOVA). **(C)** Expression of immune checkpoint receptors (ICOS and PD-1) on CD4+ or CD8+ T cells populations—N, CM, iEM, and late EM—in AML patients. Bars represent the mean ± SEM (*n* = 30; ***P < 0.001, **P < 0.01, *P < 0.05; two-tailed paired *t*-test). **(D)** Percentage of C4+ or CD8+ T cells in AML patients (either B7+, *n* = 18, or B7–, *n* = 12). Individual values are represented as points. Bars represent the mean ± SEM (*P < 0.05; two-tailed *t*-test). **(E)** Expression of immune checkpoint receptors (ICOS and PD-1) on CD4+ or CD8+ T cells populations—CM, iEM, and late EM—in AML patients (either B7+, *n* = 18, or B7–, *n* = 12) and healthy donors (HD, *n* = 4). Individual values are represented as points. Bars represent the mean ± SEM (***P < 0.001, **P < 0.01, *P < 0.05; ns, not significant; two-way ANOVA).

On an overall analysis, CD4+ T cells displayed predominantly a CM phenotype (80% of cases) and were rarely polarized as naive or effector cells. CD8+ cells displayed significantly higher late EM frequencies (*p* < 0.0001) and lower naive (*p* < 0.0001) and CM (*p* < 0.0001) cells than CD4+ cells (Figure 2A).

When comparing the CD4+ and CD8+ T cells, CD4+ T cells had significantly higher ICOS expression (*p* < 0.0001), while CD8+ expressed higher PD-1 levels (*p* = 0.0001; Figure 2B). CTLA-4 was identified at levels below 1% on all T cell populations (Table 2A).

**Table 2A:** Mean percentages of T cell maturation populations in the BM aspirate and PB of AML patients and healthy controls.

**T cells**	**T cell population**	**AML BM**	**AML PB**	**Healthy controls BM**	**Healthy controls PB**
CD4+	N	26.5%	25.7%	26.2%	33.1%
	CM	50.8%	53%	51.1%	58.2%
	iEM	12.9%	11.6%	10.4%	4.8%
	lateEM	9.5%	9.3%	11.7%	3.7%
CD8+	N	15.8%	14.6%	21%	20.5%
	CM	31.6%	30.8%	30%	30.1%
	iEM	13.5%	12.4%	13.5%	14%
	lateEM	31.8%	33.8%	32.7%	33.5%

*N, naive; CM, central memory; iEM, intermediate effector memory; lateEM, late effector memory*.

Furthermore, ICOS and PD-1 expression varied across T cell maturation subsets. ICOS had the highest levels of expression on late EM CD4+ (lateEM vs. iEM: *p* < 0.0001, lateEM vs. CM: *p* < 0.0001) and iEM CD8+ (iEM vs. lateEM: *p* < 0.0001, iEM vs. CM: *p* < 0.0001) T cells (Figure 2C).

On CD4+ cells, PD-1 expression progressively increased from the naive toward late EM cells (N vs. CM: *p* < 0.0001, CM vs. iEM: *p* < 0.0001, iEM vs. lateEM: *p* = 0.027). By contrast, on CD8+ cells, the CM and iEM subpopulations expressed the highest PD-1 levels, and late EM cells displayed the lowest levels (lateEM vs. CM or iEM: *p* < 0.0001; Figure 2C, Tables 2A,B). Additionally, our analysis showed that all the PB T cell populations mirrored the BM T cell subpopulations in both AML patients and healthy individuals (Table 2A).

**Table 2B:** Mean percentages of positivity of ICOS, CTLA-4, and PD-1 on CD4+ and CD8+ BM T cells.

**T cells**	**B7 ICR**	**AML**	**Healthy controls**
CD4+	ICOS	56.9%	57.5%
	CTLA-4	3.6%	1%
	PD-1	19.1%	15.8
CD8+	ICOS	23.8%	21.1%
	CTLA-4	1.5%	0.2%
	PD-1	27.7%	27.2%

*AML, acute myeloid leukemia; BM, bone marrow; CTLA-4, cytotoxic T lymphocyte-associated protein 4; ICR, immune checkpoint receptor; PD-1, programmed death 1*.

### The B7 Phenotype of Acute Myeloid Leukemia Blasts Is Mirrored by Distinct Modifications in T Cell Maturation and Immune Checkpoint Receptor Expression

When compared to the B7 negatives, B7-positive patients displayed significantly higher percentages of CD8+ T cells (*p* = 0.019) and lower CD4+ T cells (*p* = 0.043; Figure 2D).

Although the majority of the BM CD4+ T cells were CM cells irrespectively of the B7 phenotype, two differences were noted in B7 positive cases: (1) lower percentages of naive T cells (*p* = 0.008); and (2) higher percentages of iEM (*p* = 0.016) and late EM (*p* = 0.022) T cells. Similarly, naive CD8+ T cells were poorly represented in B7-positive AMLs (Figure 2A).

Furthermore, B7+ patients expressed higher ICOS (CM: *p* < 0.0001, iEM: *p* = 0.009, lateEM: *p* < 0.0001) and PD-1 (CM: *p* < 0.0001, iEM: *p* = 0.11, lateEM: *p* < 0.0001) levels on the effector CD4+, but not CD8+ cells (Figure 2E).

## Discussion

Similarly to solid cancers (7, 8), immune profiles in AML have been broadly described as T cell “inflamed,” in which immune cells overexpress multiple B7 ligands and ICRs, and “non-inflamed,” lacking evidence of adaptive resistance-driven immune dysfunction (9). Briefly, an “inflamed” immune profile is characterized by efficient presentation of leukemia antigens, dendritic cell activation, IFN-γ production, and the priming of leukemia-specific T cells. However, the antileukemia immune response is gradually inhibited by immune escape axes such as PD1–PD-L1, which exhaust T cells, in parallel with tumor outgrowth. By contrast, in an “immune-desert” profile, T cell priming is reduced or absent, and tolerance to leukemia is instated (7).

The “inflamed/non-inflamed” AML dichotomy might explain why patients with identical AML entities and risk profiles may have different outcomes that deviate from the initial ELN prognostic prediction (42). Recent research has demonstrated that an “inflamed” AML immune profile can predict the resistance to cytotoxic therapy but also the patient's responsiveness to immunotherapies such as ICBs or DART (9, 29, 43). However, future research is necessary to investigate how B7 immune profiling can complement the predictive ability of the ELN risk classification and guide immunotherapy.

In our study, we have identified two groups of patients based on the B7 phenotype that display T cell maturation profiles and ICR expression patterns that can be successfully reconciled with literature data regarding the immune pathogenesis of “inflamed” and “non-inflamed” cancer immune profiles.

The B7-positive group was predominant (60%) and was mostly characterized by the presence of molecules with known or most probably inhibitory role in cancer and AML in particular (B7.2, PD-L1, PD-L2, B7-H3, B7-H4) (28, 44). B7.2 and PD-L1 were the most frequently expressed checkpoint ligands, in line with previous research (30, 45). B7.1 was constantly negative. B7.2, PD-L1, PD-L2, or ICOS-L were constantly expressed across B7 signatures in several combinations that showed statistical relevance (Figure 1C), suggesting that they represent key players of AML immune evasion axes. However, statistical analysis further revealed several patterns of co-expression of the other B7 ligands that likely indicate slightly different immune evasion strategies across the various B7-positive AMLs. Thus, PD-L1 expression was correlated with B7.2 and ICOS-L positivity, but not with PD-L2, B7-H3, or B7-H4.

In complex karyotype AML-NOS, the B7 phenotype was rather restricted to isolated B7.2 expression or the co-expression of B7.2, PD-L1, and ICOS-L, which was also the most frequent B7 signature. On the other hand, normal karyotype AML-NOS cases expressed more diverse B7 ligands, including PD-L2 and B7-H4, which were statistically correlated, but also B7-H3. Interestingly, B7-H3 and B7-H4 expression was mutually exclusive, and B7-H3 expression was correlated with *NPM1* mutated AML. ICOS-L was the only co-stimulatory ligand that was identified in 40% of B7 signatures. However, its facilitating role is probably either outbalanced by the co-expression of inhibitory ligands PD-L1 or B7.2 or, according to literature data, is detrimental in itself for successful antileukemic immunity by inducing Treg expansion (46), PD-1 expression, and T cell exhaustion (47, 48). Briefly, all these B7-positive cases shared the presence of B7.2, PD-L1, or PD-L2 but had a rather heterogeneous expression of ICOS-L, B7-H4, and B7-H3, ligands that are likely involved in fine-tuning the immune escape process across different AML subtypes. In line with literature data (43), we also found that B7 ligand expression was correlated with primary refractory AML (*p* = 0.017, chi square test) (data not shown).

The B7-negative patient group was smaller (40%) and encompassed most of the AML cases with recurrent genetic anomalies, most notably with t(8,21)(q22;q22) and t(9,11)(p21;q23).

The expression of B7 ligands on AML blasts has been further mirrored by polarization differences in T cell populations and ICR expression. When the B7+ and the B7– cases were analyzed separately, it turned out that the B7+ patient cases had significantly higher cytotoxic and lower T helper cell percentages, while this ratio was reversed within the B7– group. When analyzing the maturation subsets, we were able to show that most of the CD4+ T cells fall in the CM category, unlike the CD8+ T cells where the effector subsets outnumber the central memory ones. Furthermore, B7+ patients had a significantly lower number of naive CD4+ and CD8+ T cells than the B7- patients (Figure 2A). The similarity of the CM CD4+ and CD8+ T cells in the B7+, B7–, and healthy donor groups is most probably explained by the high number of lymphocytes that do not target leukemic cells. When analyzing the effector populations, an interesting distinction could be noticed. Both iEM and late EM populations of CD4+ T cells were higher in the B7+ patients than in the B7– patients, while within the CD8+ group of cells, even though the effector populations, especially late EM, were better represented, no significant differences could be evidenced between the B7-positive and B7-negative patients. This allows us to speculate that the B7+ AML cells prove more potent in priming the CD4+ T cells and turning them into both CM and effector cells than the B7– AMLs. However, the polarization distribution of the CD8+ cells suggests that the cytotoxic lymphocytes might benefit from both T helper-dependent and independent priming.

On an overall analysis of our AML patients, setting aside the maturation polarization, the ICOS expression was predominant on CD4+ T cells, while PD-1 was higher on CD8+ cells. When compared to B7 negatives, B7-positive patients expressed higher levels of both ICOS and PD-1 on CD4+ T cells, unlike the CD8+ cells that expressed similar levels of ICOS and PD-1 regardless of the B7 positivity or negativity. Despite the significant predominance of CD8+ T cells in B7+ AML patients, it so seems that B7 checkpoint ligands are rather impacting the immunoediting of CD4+ BM T cells.

Analyzing further the ICOS and PD-1 expression on maturative subsets, we have noted a progressive increase in PD-1 expression from the naive toward the CD4+ effector compartment, while ICOS expression was highest on lateEM cells. By contrast, PD-1 expression on CD8+ cells was highest on CM, but not effector cells that displayed higher ICOS levels. Since CD4+ T cells promote the CD8+ T cell antitumor activity and prevent their exhaustion (49), we can hypothesize that CD4 T cell PD-1-mediated exhaustion precedes CD8+ cell exhaustion in B7+ AML and, more than that, is a prerequisite for CD8+ cell exhaustion.

When extending the investigation of ICOS and PD-1 expression on maturative subsets in B7-positive and B7-negative patients, we could stress further that the highest ICOS expression was present on late EM CD4+ T cells, followed by CM and iEM CD4+ T cells, and it always surpassed the ICOS expression on CD8+ T cells. Even though the level of expression was constantly higher on the cells of B7-positive patients, no statistically significant differences vs. negative ones or healthy donors emerged. PD-1 was instead always expressed at higher levels on CD8+ T cells as compared to CD4+ T cells, with the highest level reached by the CM CD8+ T cells, followed by iEM and late EM CD8+ T cells. However, the differences between B7+ and B7– patients and healthy donors were again not significant, even though iEM and late EM CD8+ T cells constantly displayed higher levels of PD1. As ICOS and PD1 binding mediate activating, respectively, inhibitory signals, these results might suggest that, irrespectively of B7 expression on AML blasts, CD8+ T cells are more prone to PD-1-mediated apoptosis, while their CD4+ counterparts are more susceptible to activation, but also to activation-induced cell death. Furthermore, the presence or absence of the B7 molecules on the AML blasts seems to have a minimal impact in influencing the levels of expression of ICOS and PD-1 on the T cell surfaces.

Finally, we have aimed to harmonize our data with the models of AML immune pathogenesis with “inflamed” and “non-inflamed” microenvironments [reviewed in Davidson-Moncada et al. (9)]. Thus, in our study, it was the intermediate and adverse risk AML cases which were frequently B7 positive (78.2%), as well as *NPM1* mutated AML, that displayed features holding indirect evidence of an “inflammatory” microenvironment, including the expression of mainly inhibitory B7 ligands, correlated with higher percentages of CD4 effector cells, less CD4+ and CD8+ naive T cells, as well as higher ICOS and PD-1 expression. *NPM1* mutated AML also presented effector differentiation of CD8+ PD-1+ T cells, which is likely a feature of immune exhaustion. The rather inflammatory polarity of the immune microenvironment in *NPM1* mutated AML is further supported by research that revealed that *NPM1* mutation generates immunogenic peptides (50), an IFN-γ-driven T cell response (51) and is correlated with B7-H3 and PD-L1 expression (32, 52). These correlations are relevant since literature suggests that “inflamed” AML could benefit from ICB or even synergistic DART/ICB approaches (9, 43).

By contrast, B7-negative AML was characterized by higher percentages of naive CD4+ and CD8+ T cells and lower ICR expression and was more prevalent in AML with t(8,21)(q22;q22) but also in AML harboring the t(9,11)(p21;q23), an entity with rather low immunogenicity, as shown by previous research (53, 54). Thus, these non-inflamed, low immunogenic AML types are likely less capable of priming T cells and mounting antileukemia immune responses. Therefore, B7-negative AML should be approached differently by adoptive cell transfer (chimeric antigen receptor T cells), leukemia peptide vaccines, or strategies that augment tumor cell immunogenicity and convert “non-inflamed” LMEs to inflammatory ones, such as hypomethylating agents (9, 15).

It is most clear that further research is needed to improve the characterization of the T cell composition and immune checkpoint landscape of the BM microenvironment. Recently, a comprehensive study conducted by Williams et al. (33) addressed the frequencies of BM T cell populations and their ICR expression as well as the expression of B7 checkpoint ligands on tumor cells of 39 newly diagnosed and 68 relapsed AML patients and correlated them with standard AML prognostic factors. The authors showed that immune exclusion, i.e., the absence of T cell BM infiltration, is not a feature of AML, since BM T cell percentages were similar to the age-matched HD, a finding that has been replicated by our study. However, AML patients had slightly higher Treg percentages when compared to healthy donors. The study of Williams et al. (33) shows that the BM microenvironment has inflammatory features in a subgroup of patients since the numbers of effector helper and cytotoxic T cells were increased. Furthermore, CD4+ cells overexpressed the co-stimulatory molecules OX40 and ICOS, and PD-1 had higher expression percentages levels on both CD4- and CD8-positive T cells when compared to the control group. The differences in ICOS and PD-1 expression on CD4+ and, respectively, CD8+ cells in our study also suggest that helper and cytotoxic T cell exhaustion is a process regulated by subtle differences in the expression of co-stimulatory and co-inhibitory immune receptors. We consider this finding important for the design of future combination immunotherapies since successful ICB relies on the T helper-assisted generation of cytotoxic antitumor effector cells (7). Williams et al. (33) also show that T cell exhaustion is likely a multistep process, with latter stages of exhaustion co-expressing PD-1 and TIM-1 (T cell immunoglobulin and mucin domain containing-3) or LAG-3 (lymphocyte-activation gene 3) and indicating the presence of more antigen-experienced T cells in the BM milieu and an inflammatory environment. Regarding the expression of B7 ligands on AML cells, Williams et al. (33) correlated PD-L1 expression with *TP53* mutation and adverse karyotypes. In our similarly sized group of newly diagnosed AML patients, we were able to confirm this association between the expression of B7 ligands, including PD-L1 and the adverse-risk ELN patient subgroup.

Williams et al. (33) did not present survival data since the patients had received various treatment modalities in different clinical trials. Although we found a statistically relevant detrimental effect of B7 expression on overall survival and correlated B7 expression with the presence of primary refractory AML (data not shown), we consider that these data require validation on a larger patient cohort since primary refractory AML patients represent a population with a particularly poor prognosis which might be independent of B7 expression.

Our study has obvious limitations, including the deliberate simplification of the leukemia immune biology, which depends on many other factors, such as functional T helper polarization (Th1, Th2, Th17), NK cells, macrophages or myeloid derived suppressor cells, and a relatively small patient group made of exclusively newly diagnosed AML cases however reflecting the heterogeneous AML patient population and confirming several conclusions emerging from the study of Williams et al. (33).

Our data provide a more in-depth insight on the immune biology of AML. The B7 expression and antileukemic immunity in general are continuously regulated by a plethora of factors, including tumor-independent ones (7, 15). Hence, the interaction between immune effectors and AML blasts should be regarded as a dynamic process and the B7 phenotypes of these cells might be subjects of change across the longitudinal evolution of the patients (9). For example, a patient with favorable risk AML might present with an immune desert B7-negative phenotype at diagnosis, which, at relapse, might convert to an inflammatory B7-positive one. Thus, it is more likely that independently of the ELN cytogenetic risk of AML, the B7 phenotype would rather indicate which immunotherapy approach is more suitable for the patient's type of immune dysfunction at that specific moment than assist practitioners in refining the long-term prognosis of AML patients.

All in all, our results reinforce the concept that this genetically heterogeneous disease has distinct and versatile patterns of antitumor immune response that depend on factors beyond the intrinsic genetic traits of the tumor cells. This finding is particularly relevant since new AML drugs are being rapidly developed and immune profiles emerge as a powerful biomarker in guiding and personalizing the new immunotherapy approaches.

## Conclusions

Gene expression profiling of the leukemia immune microenvironment is laborious, time-consuming, and not widely available. Hence, alternative markers for a faster and less expensive evaluation of the AML immune landscape are needed. In this paper, we have demonstrated that B7 checkpoint receptor and ligand profiling by flow cytometry is able to generate relevant data.

In our study group, in more than half of the cases, the AML blasts expressed B7 molecule signatures or isolated B7 ligands. Regardless of the B7 combination, a co-inhibitory B7 molecule, such as B7.2, PD-L1, or PD-L2, was always present. B7 molecule expression was more frequent in intermediate and adverse ELN risk AML (78.2%) when compared to favorable risk cases (28.5%). Finally, B7 positivity was correlated with primary refractory AML and reduced overall survival.

B7-positive AMLs displayed a predominance of effector BM CD4+ T cells with significantly higher levels of ICOS and PD-1. B7-negative AMLs were characterized by a significant predominance of naive CD4+ and CD8+ T cells and lower CD4+ T cell ICOS and PD-1 levels.

As B7 ligands and their counterpart T cell receptors are regarded as indirect indicators of an IFN-driven “inflammatory” microenvironment, we can thus hypothesize that the B7 immune profiling of AML blasts and T cells could serve as a useful biomarker to rapidly discriminate between AMLs with “inflamed” vs. “non-inflamed” microenvironment. Rather than defining an AML subtype in itself, the B7 phenotype more likely offers a momentary perspective of the versatile interaction between leukemia and immune cells that could predict resistance to standard chemotherapy and could guide personalized immunotherapy.

We have undoubtedly entered a new therapeutic era in AML, where new and effective drugs are being rapidly designed, but the progress of immunotherapy clinical trials has a tendency to outpace our understanding of leukemia immune biology. The immune profiling of the tumor microenvironment will likely have a major impact on future clinical trial design, drug development, and integration of personalized immunotherapy in current therapeutic strategies.

## Data Availability Statement

The datasets generated for this study are available on request to the corresponding author.

## Ethics Statement

The studies involving human participants were reviewed and approved by Ethics Committee, Grigore T. Popa University of Medicine and Pharmacy, Iasi, Romania. The patients/participants provided their written informed consent to participate in this study.

## Author Contributions

IA, MZ, and PC are responsible for the study design. IA, AD, and CD were involved in the clinical management of the patients. MZ, II, and AS performed the immunophenotypic, molecular, and cytogenetic analyses. MP performed the statistical analysis and contributed to the graphical illustrations. IA and PC wrote the manuscript. All the authors were involved in the revision of the manuscript.

### Conflict of Interest

The authors declare that the research was conducted in the absence of any commercial or financial relationships that could be construed as a potential conflict of interest.
