# Supplementary material for: B7-Positive and B7-Negative Acute Myeloid Leukemias Display Distinct T Cell Maturation Profiles, Immune Checkpoint Receptor Expression, and European Leukemia Net Risk Profiles
Source: Front Oncol. 2020 Mar 13;10:264. doi: 10.3389/fonc.2020.00264 (PMC7082324; doi:10.3389/fonc.2020.00264)
Supplement: Supplementary file 1 [file Table_1.docx]

**Supplementary Table 1.**

Fluorochrome-conjugated monoclonal antibodies that were used in this study.

|  | mAb | Fluorochrome | Clone | Manufacturer |
| --- | --- | --- | --- | --- |
| 1. | Anti-CD45 | Brilliant Violet (BV) 510 | HI30 | BioLegend^TM^ |
| 2. | Anti-HLA-DR | APC-Cy7 | L243 | BD biosciences^TM^ |
| 3. | Anti-CD117 | BV 421 | 104D2 | BioLegend^TM^ |
| 4. | Anti-CD34 | BV421 | 561 | BioLegend^TM^ |
| 5. | Anti-CD80 | Alexa Fluor (AF) 488 | 2D10 | BioLegend^TM^ |
| 6. | Anti-CD86 | PerCP Cy5.5 | IT2.2 | BD Biosciences^TM^ |
| 7. | Anti-CD 273 | Allophycocyanin (APC) | 24F.10C12 | BioLegend^TM^ |
| 8. | Anti-CD274 | Pe-Cy7 | 29E.2A3 | BioLegend^TM^ |
| 9. | Anti-CD275 | Phycoerythrin (PE) | 9F.8A4 | BioLegend^TM^ |
| 10. | Anti- CD276 | PE | DCN.70 | BioLegend^TM^ |
| 11. | Anti- B7-H4 | APC | MIH43 | BioLegend^TM^ |
| 12. | Anti- CD3 | FITC | SK7 | BioLegend^TM^ |
| 13. | Anti-CD8 | BV510 | SK1 | BioLegend^TM^ |
| 14. | Anti- CD4 | BV510 | SK3 | BD Horizon^TM^ |
| 15. | Anti-CD45RA | APC-H7 | 5H9 | BD Pharmingen^TM^ |
| 16. | Anti- CD27 | BV421 | M-T271 | BD Horizon^TM^ |
| 17. | Anti- CD28 | PerCP Cy5.5 | L293 | BD biosciences^TM^ |
| 18. | Anti-CD152 | APC | L3D10 | BioLegend^TM^ |
| 19. | Anti- CD278 | PE/Cy7 | C398.4A | BioLegend^TM^ |
| 20. | Anti- CD279 | PE | EH12.1 | BioLegend^TM^ |
